# What Works (or Doesn’t) in Return to Work after Physical Injury? A Qualitative Study on the Perspectives of Trauma Patients and Health Care Professionals on Barriers and Facilitators in Return to Work

**DOI:** 10.1007/s10926-025-10309-z

**Published:** 2025-07-05

**Authors:** E. de Groot, A. M. Hermans, M. A. C. de Jongh, R. E. Geuze, I. M. van Dongen, S. Hommes, R. D. Vromans, E. Krahmer, T. Houwen, K. M. E. Janssens, M. C. W. Joosen

**Affiliations:** 1 Tranzo, Scientific Center for Care and Wellbeing, Tilburg School of Social and Behavioral Sciences, Tilburg University, P.O. box 90153, 5000 LE Tilburg, the Netherlands; 2 Netwerk Acute Zorg Brabant, Elisabeth-TweeSteden Ziekenhuis, Tilburg, the Netherlands; 3 Department of Orthopaedics, Elisabeth-TweeSteden Ziekenhuis, Tilburg, the Netherlands; 4 De Nieuwe Arts - Occupational Health Service, Amsterdam, the Netherlands; 5 Department of Communication and Cognition, Tilburg School of Humanities and Digital Sciences, Tilburg University, Tilburg, the Netherlands; 6 Department of Surgery, Elisabeth-TweeSteden Ziekenhuis, Tilburg, the Netherlands

**Keywords:** Trauma, Return to work, Vocational rehabilitation, Follow-up care, Recovery

## Abstract

**Purpose:**

Return to work (RTW) after physical injury may be challenging. This study aims to gain insight into barriers and facilitators in RTW, using a multi-stakeholder perspective from trauma patients with diverse injuries and health care professionals (HCPs).

**Methods:**

Three focus groups (*n* = 13) and four interviews were conducted with patients who sustained an extremity injury, spinal injury, or traumatic brain injury. Four focus groups (*n* = 19) were conducted with HCPs (e.g., occupational physicians, trauma surgeons). Data were transcribed verbatim and thematically analyzed for patients and HCPs separately.

**Results:**

Both patients and HCPs emphasized that RTW can be influenced by the extent to which core work tasks are affected by the injury. Barriers in RTW related to lack of RTW follow-up care, limited consultation time, and lack of support in the work environment. Shared facilitators were found in work not affecting the injury, and when adequate support is provided by HCPs and the work environment. While patients and HCPs shared similar ideas, perspectives diverged on RTW follow-up care, provided by HCPs in hospital. HCPs in hospital perceive physical recovery as their core task instead of RTW follow-up care, which is perceived by patients to detract attention to life after injury.

**Conclusion:**

According to patients and HCPs, regardless of the injury, the combination of injury type and job type influences RTW. Across injuries, receiving RTW follow-up care from HCPs in hospital was often missed, while perceived to facilitate RTW. Tailored RTW information could be useful to patients in resuming life after injury.

## Introduction

Physical injury (or trauma), for example caused by an accident, can significantly impact people’s ability to work [1]. While most trauma patients return to work (RTW) relatively quickly, a substantial group experiences long-term health problems which can result in diminished work productivity, prolonged absence, or even unemployment [2]. Complete, partial, or temporary inability to RTW due to injury also has financial ramifications for society [3]. On an individual level, loss of work is associated with poorer quality of life [4], since having paid employment is linked to self-confidence, financial independence, and a sense of purpose and identity [5, 6]. In fact, work is found to be an important contributor to recovery, and thus enhances quality of life [7–9]. To facilitate RTW, follow-up care and guidance can be provided (for example by an occupational physician), to continue monitoring and offering support after primary treatment for injuries has ended.

However, follow-up care focused on RTW is often provided late, namely *after* physical rehabilitation, or is not provided at all [10–15]. Providing information on RTW earlier in the rehabilitation phase may contribute to a more timely and sustainable RTW. In order to be able to provide relevant information, it is important to explore what is perceived to be hindering and/or facilitating in RTW. Previous studies looking into barriers and facilitators in RTW after injury often include one perspective, usually coming from patients [6, 7, 13, 16–19], and to a lesser extent from health care professionals (HCPs) [20, 21]. However, by including both perspectives, which is novel in this domain, a more complete understanding of what hinders or facilitates the RTW process after physical injury is gained.

Additionally, while previous studies have provided insight into these barriers and facilitators, they often included only one type of injury, such as traumatic brain injury (TBI) or spinal injury (SI) [16, 17, 20, 22–25], whereas this study looks beyond trauma types. Studies including a broader range of trauma types are lacking yet needed, as factors transcending injury type and/or severity may play a role in RTW, like presence or absence of a support system, or a supportive or unsupportive working environment [26]. This perspective is reinforced by Cancelliere et al. [27], who performed an umbrella review identifying common prognostic factors for RTW across various health conditions. This comprehensive study found that people with musculoskeletal disorders, cancer, cardiovascular disease, and mental health problems share common RTW barriers and facilitators. They highlight the need for further research into these factors in conditions other than musculoskeletal disorders, which have received the most attention to date. A similar approach can be found in psychiatry, a domain which is shifting from thinking in terms of categorical diagnoses and their underlying symptoms, to symptoms which are cutting across diagnoses, also called the transdiagnostic approach [28]. In this light, while barriers and facilitators in RTW may be well researched per type of injury in single studies, it remains unclear what the overarching hindering and facilitating factors in RTW are *across* injuries. These factors could be thought of *transfactors*.

Still, depending on the occupation involved, barriers in RTW may differ within injury types. That is, a broken ankle may be more problematic for a construction worker than a cleric. In that aspect, providing tailored information on reintegration after physical injury could be helpful in patients’ preparations to RTW. Moreover, combining the perspectives of patients with diverse injuries and perspectives of HCPs from various fields may provide deeper insights into this process.

Therefore, the goal of this study is to identify the barriers and facilitators to RTW after injury using a multi-stakeholder perspective by looking at the experiences of a heterogeneous trauma population and the perceptions of a diverse range of HCPs involved with trauma patient care.

## Methods

### Study Design

A qualitative focus group and interview study was conducted using a thematic content analysis to explore barriers and facilitators trauma patients and HCPs experienced in RTW. Focus groups were conducted with (1) patients and (2) HCPs, followed by in-depth semi-structured interviews with patients to enrich the data obtained. Focus groups were expected to elicit interaction among participants, potentially leading to recognition or discussions [29]. The study was designed and reported according to the COmprehensive consolidated criteria for REporting Qualitative research (COREQ) [30], and evaluated and approved by The Ethics Review Board of Tilburg University (TSB_RP435) and by the Board of Directors from the Elisabeth-TweeSteden Ziekenhuis.

### Recruitment

#### Patients

Patients were recruited via a level 1 trauma center in the South of the Netherlands, where patients were hospitalized after injury for at least one night. They were selected from electronic patient records and the trauma registry of the hospital (TH and a data manager). To capture a broader trauma population, SI, TBI, and isolated fractures (IFs, one or more fractures of the upper or lower extremities) were included. While TBI may be perceived as distinct from fractures, it is a form of traumatic injury. Moreover, TBI is an underexplored subgroup despite its large prevalence in the trauma population. Therefore, exploring shared experiences across these injury groups is perceived to be valuable.

Targeted participants were (1) of working age (18–67 years) at the time they got injured; (2) admitted to the hospital between 9 months and 5 years ago (to ensure sufficient recovery for RTW while minimizing recall bias); and (3) sustained one of the following injuries:

- IFs;
- mild-to-moderate TBI, with an Abbreviated Injury Scale (AIS) of ≤ 3 and secondary injuries AIS ≤ 2 (AIS is a severity scoring system, ranging from 1 to 6, to classify the severity of physical injuries) [31]; and
- SI AIS ≤ 5 and secondary injuries AIS ≤ 2.

Patients eligible according to these criteria were invited to participate via email. In total, 253 patients were invited (104 IF, 88 TBI, 61 SI), of which 41 signed up. Upon providing digital informed consent, patients answered a short demographic questionnaire, enabling purposive sampling to ensure maximal variation in terms of age, gender, and occupation. Variation in occupation was determined by first balancing for age and gender, followed by selecting jobs with high and low physical activity demands. This resulted in 17 participating patients. Eligible patients who were unavailable on the dates for the focus groups or who preferred an interview were identified as potential interviewees. The interviewers and focus group facilitators were not involved in patient care and had no prior relationship with the participating patients. Patients did not have knowledge about the researchers involved. Patients received a voucher of 40 euros for their participation.

#### HCPs

HCPs across primary care (e.g., general practitioners, physical therapists, and occupational therapists), secondary/inpatient care (e.g., orthopedic surgeons and rehabilitation doctors), and occupational care (e.g., occupational physicians) were recruited via email through the team’s network. Moreover, calls for participation were sent to specific associations of professions involved with trauma patients. HCPs were eligible if they had worked with trauma patients, irrespective of the time since their involvement. In total, 27 HCPs signed up and provided digital informed consent. Next, they answered a short demographic questionnaire, and purposive sampling was used to ascertain professional diversity within the focus groups, resulting in 19 participating HCPs who received a small token of appreciation (a chocolate bar) for their time and effort.

### Data Collection

Focus groups and interviews for both patients and HCPs were conducted via Microsoft Teams between May and September 2023. Focus groups lasted approximately 1.5 h, and were facilitated by at least two members of the research team (AMH, female, PhD, with expertise in social sciences and qualitative methods, EdG, female, MSc, with expertise in communication and information sciences and qualitative methods, KJ, female, PhD, with expertise in psychology, or MJ, female, PhD, with expertise in health sciences and qualitative research). All interviews were conducted by one researcher (EdG) and lasted between 30 and 60 min. Field notes were taken during focus groups and interviews. Prior to the focus groups and interviews, participants received technical instructions on how to participate. All conversations were audio and video recorded, with permission of the participants, using the recording feature provided by Microsoft Teams.

#### Patients

A semi-structured topic guide was developed for the focus groups as well as for the interviews, based on relevant literature [13, 32]. The guide comprised two parts: (1) experiences with maintaining, losing, and/or returning to work; and (2) experiences with and needs for follow-up care and guidance in RTW (see Appendix 1). The topic guide was pilot tested, after which some questions were rephrased.

Three online focus groups took place with four to five patients each (*N* = 13). Patients were divided into injury-specific (IFs, TBI, or SI) groups to increase the likelihood that patients could relate to one another. Following the focus groups, four in-depth interviews were held.

#### HCPs

A semi-structured topic guide was developed in which two topics were discussed, namely the HCP’s perceptions of (1) care, follow-up care, and guidance provided to trauma patients; and (2) hindering and facilitating factors experienced by trauma patients in RTW (see Appendix 2). Some questions were altered after pilot testing for clarity purpose. Four focus groups with three to six HCPs from different disciplines were conducted, after which no new insights were found.

### Data Analysis

Recordings were transcribed verbatim by Noto Services, a professional transcription agency. Any identifiable information was left out. Transcripts were analyzed thematically according to the six-phase process by Braun and Clarke [33] using ATLAS-ti.23. The topic guides were used as a framework, yet inductive coding of information relevant to the aim of the research also took place.

The coding processes for patients and HCPs were similar in nature. Coding was carried out by at least two researchers (AMH, EdG, KJ). First, one focus group transcript for patients and one focus group transcript for HCPs were open coded independently. The open codes were then compared, discussed, and grouped together, resulting in one preliminary axial coding scheme for patients, and one for HCPs. These schemes were used to analyze the second focus group transcript for patients and HCPs, and two patient interview transcripts (EdG). Revisions were made to the axial coding schemes upon identification of additional codes, which were discussed among the researchers (AMH, EdG, KJ). The remaining transcripts and the first ones were (re)coded according to the revised coding schemes (EdG). During the debriefing among the research team and summarization of preliminary themes, no new insights contributing to answering the research questions were found, indicating data saturation [34]. Next, related axial codes were merged by one researcher (EdG) and organized into main themes and subthemes for both patients and HCPs. These candidate themes were discussed with the research team (AMH, EdG, KJ, MJ, MdJ), after which themes and subthemes were rephrased and revisited until consensus was reached. Lastly, the clinical and practical implications of the analysis were discussed with a clinical practitioner (RG).

## Results

Barriers and facilitators to RTW after injury according to patients will be discussed first, followed by barriers and facilitators according to HCPs. Results will be presented in multiple (sub)themes, preceded by a short introduction to each theme.

### Barriers and Facilitators to RTW After Injury According to Patients

The recruited patients were nine females and eight males with an average age of 47 years. Most patients sustained their injury 2–3 years ago. There was an equal representation for each type of injury (*n* = 6 IF, *n* = 6 TBI, and *n* = 5 SI). Table 1 provides a full overview of the sample.

**Table 1:** Demographics of patients

P	Gender	Age	Injury	Date of Injury	Employed	Occupation	RTW	Source
1	F	20	SI	2021	Employed	Parcel deliverer/Former Student*	FT	FG
2	F	66	SI	2022	Self-Employed	Childminder agency	PT	FG
3	M	68	SI	2020	Self-Employed	Floor installer	PT	FG
4	M	60	SI	2020	Employed	Car mechanic	No	FG
5	M	54	IFs	2021	Self-Employed	Machine mechanic	FT	FG
6	F	29	IFs	2021	Employed	Elementary teacher	FT	FG
7	F	61	IFs	2020	Employed	Administrative	FT	FG
8	M	32	IFs	2021	Employed	Backoffice	FT	FG
9	F	58	IFs	2021	Employed	Teacher/care coordinator	FT	FG
10	M	35	TBI	2019	Employed	Engineer	FT	FG
11	F	22	TBI	2019	Employed	Waitress/Student	PT	FG
12	M	63	TBI	2020	Employed	Administrative	No	FG
13	M	65	TBI	2019	Employed	Vet	FT	FG
14	F	54	SI	2020	Self-Employed	Driving instructor	PT	I
15	M	37	TBI	2021	Employed	Socio therapist	FT	I
16	F	49	IFs	2021	Employed	Administrative	FT	I
17	F	28	TBI	2020	Employed	Speech therapist	PT	I

*F* female, *M* male, *IFs* isolated fractures, *SI* spinal injury, *TBI* traumatic brain injury, *FT* fulltime, *PT* parttime, *FG* focus group, *I* interview

*This participant was a student at the time of sustaining the injury and now works as a parcel deliverer

The barriers and facilitators patients identified were divided into three distinct themes: (1) extent to which the injury has impact on work tasks and finances; (2) timely, adequate, and protective RTW follow-up care provision by HCPs; and (3) importance of managing expectations and adjustments in work and support. Themes were divided into subthemes, and illustrated with a participant’s quote (for more quotes, see Table 2).

**Table 2:** Thematic map with themes and subthemes, illustrated with patients quotes

Theme	Subthemes	Quotes
Extent to Which the Injury Has Impact on Work Tasks and Finances		“I was in the policy department, so that made a difference. I could decide for myself how many hours I wanted to work or not. But well, within, yeah, I think two, three, at most four weeks, I was back to working full-time.” (P13, TBI) “As a self-employed person, you can insure yourself, but you’ll never get the benefits to cover all your expenses. So, at some point, your savings start to run out after about seven months, especially with two teenagers who are also studying. So yeah, you have to do something […] It’s also about pushing your limits.” (P5, IFs)
		“I really needed money for my care and all the fixed costs I had, but I couldn't work, and the money I received from my work was less than you normally get. So for me, at that moment, my side job income was 150 euros. While the travel costs were already 100 or more euros, so there were a lot of factors that played a role for me, which made me feel kind of forced to go to work immediately.” (P11, TBI)
Timely, Adequate, and Protective RTW Follow-Up Care Provision by HCPs	(RTW) follow-up care and guidance perceived as inadequate	“What I consider to be the only limitation, like I said with the specialists actually, it was all about the complaint but not about the consequences of that and how you have to proceed with it and […] yeah, just see what you can do and figure it out.” (P7, IFs) “It was really like ‘here are crutches, and here’s a wheelchair, and just figure it out yourself’, you know, that kind of misery.” (P9, IFs) “That occupational physician who took every opportunity to increase my hours again, because, well, he’s of course there for me, but in the end, he’s really there for the employer, so, yeah. I really notice it, that as soon as you show a little bit of positivity, they grab onto it and say ‘Go ahead, you can do it’. In hindsight, I felt they took advantage of that by saying ‘oh great, you can work a few hours more.” (P11, TBI)
	Suitable timing of follow-up care provision on RTW	“I would say about a month after discharge from the hospital, something like that. Because then you're really engaged in your recovery process, and you're not just lying in bed anymore; you start to see what you can do, what you can't do.” (P17, TBI)
	HCP as protective barrier in RTW	“I really wanted to finish school but if you [physically] can’t, you can’t. I really wanted to continue but if someone [physical therapist] then tells me like ‘it’s okay if you don’t just now, how much does a year matter in 50 years’. Yeah, well, nothing. Yeah, that was nice to hear, that I could think about myself for a while.” (P1, SI)
Importance of Managing Expectations and Adjustments in Work and Support	(No) effective work adjustments	“They [employer] said ‘We’ll arrange a stool on wheels’, but then you still need your knees when you want to move, and they’re positioned in a weird angle because you’re on that [stool] […] And he [employer] insisted ‘We took every measurement necessary to keep her at school [workplace]’, which put him [employer] at odds with the occupational physician who said ‘[RTW] isn’t possible yet.’” (P6, IFs)
	(Unrealistic) RTW expectations and (lack of) support from others	“At work, they wanted me to work fulltime again. They said ‘take it easy, but hey, you’re not going to school so than you should be able to work’.” (P11, TBI) “What I found really difficult was that my employer saw me walking with those 1000 steps a day, which went fine. But that was just for basic things, walking to the bathroom in the evening, walking to the kitchen and standing to cook, and then you’ve done so many steps. And when I had an appointment with [employer] in the morning at 9:30 to talk about my progress, I’d show up pretty cheerful and fairly quickly with two crutches, then later with one crutch, instead of being in a wheelchair. So he thought ‘Oh, that’s great, we can get her back in front of the class quickly’, and with all those [staffing] shortages, that was of course his biggest wish.” (P6, SI)
		“They [colleagues] have only helped me, and my boss, yeah, he's always been involved and regularly checks in as well.” (P4, SI) “At one point, when my direct colleague was on vacation, my manager picked me up a few times from home because I couldn’t drive [to work].” (P16, IFs)

#### Theme 1: Extent to Which the Injury Has Impact on Work Tasks and Finances

Physical injuries may have impacted people differently. Sometimes injuries had substantial impact, while other times the impact seemed smaller. This depended on factors like the type of job and the severity of the injury. Some patients, for which their injury had little impact on their work tasks, perceived limited constraints to RTW (e.g., the driving instructor with SI wearing a back brace in the car). Within a couple of weeks these patients were back at work, with or without work adjustments. Additionally, patients who had a quick physical recovery found it relatively easy to RTW.

“Functioning went quite well already. With a broken leg you don’t have to adjust a lot [at work].” (P16, IFs)

When the injury affected the patients’ work tasks in such a way that they were temporarily or permanently unable to work, it could impact their finances. Particularly self-employed patients perceived these financial difficulties, as was the case for a floor installer whose ankles were broken; not being able to work and having no insurance meant there was no income, or less than before the injury. These feelings of financial pressure seemed to facilitate the drive to RTW faster than was actually healthy, instead of taking the time to recover properly, which ultimately hindered their recovery.

“I do think that if I had been employed, I would have said: ‘Sorry, but today it's not going to work.’ Now [being self-employed], you have much more of a drive to say ‘I have to [work], period’.” (P14, IFs)

Moreover, two patients who were still in college and worked parttime to cover expenses and leisure, experienced financial pressure in their RTW. They explained their expenses rose because of their injury, yet their income decreased or ceased altogether because they were unable to work.

“When I had my accident, I didn’t have a steady job, and I was in school. So I had a lot of expenses, but I wasn’t earning any money at all. Now, I’m working really hard to pay everything off, so I can start school next year without any debt.” (P1, SI)

#### Theme 2: Timely, Adequate, and Protective RTW Follow-up Care Provision by HCPs

The (in)adequacy of the follow-up care in RTW provided by HCPs in general, if provided at all, seemed to play an important role in the experience of the RTW process.

*(RTW) Follow-up Care and Guidance Perceived as Inadequate *Almost all patients mentioned receiving no or limited RTW follow-up care. Some deemed the follow-up care to be insufficient, particularly as there was limited personalized care. That is, patients felt that their personal situation was not taken into account, resulting in generalized follow-up care. For instance, follow-up care mainly focused on functional recovery, and little to no attention was given to a patient’s life after injury, including work. Furthermore, the prognoses on recovery or RTW were perceived to be inaccurate and not tailored to the patient. HCPs in hospital were frequently mentioned to fall short in these areas.

“From the very beginning, […] I’ve thought ‘Hey, I missed something after my return [to work]’. You see, in my opinion, follow-up care was minimal. Or non-existent. Actually, just not there at all.” (P15, TBI)

Some patients even felt skeptical about the offered guidance. For instance, patients perceived that occupational physicians exerted pressure to RTW, serving the interests of employers, who would benefit from early reintegration, not the employees, who would benefit from support in their recovery.

*Suitable Timing of Follow-up Care Provision on RTW *Some patients stressed that RTW follow-up care by professionals (e.g., occupational physician, physical therapist) was not always needed for patients who had a quick recovery, who had jobs that were not greatly affected by their injury, or who made arrangements concerning work themselves.

For those who expressed the need for RTW follow-up care, the timing of introducing RTW discussions seemed essential. Some patients mentioned that work, or RTW, was not on their minds right after sustaining the injury. Information about work was more welcome at a later stage, e.g., after discharge from the hospital, when it became clearer what one can and cannot do.

“At first, I really wasn’t focused on my work because I was focused on myself.” (P2, SI)

*HCP as Protective Barrier in RTW *Having HCPs as protectors was mentioned as a facilitator in sustainable RTW. For example, several patients felt supported by the occupational physician or health and safety officer when their employer exerted pressure to RTW. Patients also indicated they needed protection from themselves to prevent a premature RTW (e.g., because of overestimating their own abilities to work, or feeling internal pressure to RTW as quickly as possible because of the feeling their absence would burden colleagues). These patients overstretched, resulting in a setback or having to recuperate for some days. The physical therapist and occupational therapist were frequently mentioned as a great support to prevent overstretching.

“If it wasn’t for my occupational physician, I think things would have gotten out of hand. I would have just kept working. He was the one who had to convince everyone, including myself, that this was the limit and no further.” (P6, IFs)

#### Theme 3: Importance of Managing Expectations and Adjustments in Work and Support

The extent to which colleagues or employers are (not) understanding and supportive during reintegration seemed to have an influence on RTW.

*(No) Effective Work Adjustments *Not being provided with suitable adjustments in the work environment was perceived to hinder recovery and therefore hinder RTW. The lack of suitable adjustments was due to various reasons. For instance, core tasks cannot be changed or altered; e.g., heavy lifting is part of the job as a parcel courier. Adjustments that did not suffice or did not have the desired positive effect led to setbacks in recovery, which affected RTW. Like the elementary teacher with broken knees, who was provided a stool on wheels by her employer to move around, but had to push it with her knees, which hindered recovery and worsened her condition.

Contrastingly, patients indicated that it was helpful to make functional adjustments due to the injury in relation to work tasks or schedules. Working fewer hours, for example, contributed to RTW. During the RTW process, patients appreciated having a professional such as the occupational therapist and/or a reintegration coach looking over their shoulder and, in joint consultation, making changes to their work to fit their abilities. These changes did not relate to work tasks per se but rather to how work was executed and scheduled, with the goal to increase the chances of sustainable RTW.

“Normally, you’ll plan eight or nine [clients] in a row, then you’ll have lunch, and then you’ll plan another seven. I really have to make smaller blocks. So I’ll do blocks of four or five [clients]. So, there are a lot more breaks during my day.” (P17, TBI)

*(Unrealistic) RTW Expectations and (Lack of) Support From Others *Apart from the self-employed patients, about half of the patients expressed that expectations of the work environment were sometimes too high, and they felt pressured to RTW as soon as possible. These participants experienced a lack of support. Some employers, for example, exerted pressure to work as soon as patients’ abilities seemed to improve. Furthermore, patients with TBI often mentioned feeling judged by others, like their employer, because of the incongruence in the way they feel internally and their (often ‘normal’) external appearance, which caused self-doubt.

“I also have a little boy who wakes up at night too. Luckily, my wife handles all of that. If I had to do that as well, I think working would be a completely different story. These are things you don’t see on the outside. And people only know the story on a very superficial level, and they say ‘Yeah, you're back and working, so everything's fine’. No, there are underlying things that are always there, and I think they'll always remain.” (P10, TBI)

Although many patients experienced pressure to RTW, others experienced a lot of support from employers, colleagues, business relations, and if applicable, school. These patients perceived no pressure to RTW. In fact, patients indicated colleagues were often willing to take over tasks, clients, or other work. This kind of support was perceived as helpful for RTW.

“On Thursday I had a scheduled [driving] exam. A colleague took over, so that particular student could take the exam after all.” (P14, SI)

### Barriers and Facilitators to RTW After Injury According to HCPs

Participating HCPs were nine females and ten males, with an average age of 46 years. HCPs had mixed backgrounds and professions (e.g., trauma surgeon, occupational physician). Table 3 provides a full overview of the sample.

**Table 3:** Demographics of professionals

P	Gender	Age	Profession
18	F	54	Occupational Physician
19	M	48	Neurologist
20	F	50	Allied Healthcare Advisor
21	F	39	Psychologist
22	M	63	General Practitioner
23	F	42	Occupational Physician/former Orthopedic Surgeon
24	F	44	Insurance Physician
25	F	50	Rehabilitation Physician
26	F	57	Occupational Therapist
27	M	30	Trauma Surgeon
28	F	57	Psychologist
29	M	38	Physical Therapist in Primary Healthcare
30	M	31	Physical Therapist in Secondary Healthcare
31	M	43	Occupational Physician
32	M	40	Orthopedic Surgeon
33	M	60	Rehabilitation Doctor
34	M	52	Occupational Physician
35	F	57	Occupational Therapist
36	M	33	Physical Therapist in Secondary Healthcare

^*F* female, *M* male^

Barriers and facilitators for patients’ RTW after injury were identified by HCPs, and related to four main themes: (1) extent to which the injury affects work, self-confidence, and finances; (2) the (lack of) attention on work in a clinical sector which is focused on injury and recovery; (3) motivation to RTW depends on patients’ attitude towards abilities and gains; and (4) importance of receiving or lacking support from employers and colleagues. Themes are divided into subthemes, and supported with a participant’s quote (for more quotes, see Table 4).

**Table 4:** Thematic map with themes and subthemes, illustrated with quotes from HCPs

Theme	Subthemes	Quotes
Extent to Which the Injury Affects Work, Self-Confidence, and Finances		"The plasterer, for instance, with two broken ankles and arthrodesis on both sides, is no longer able to climb that ladder afterwards [the injury], so to speak.” (P24).
The (Lack of) Attention on Work in a Clinical Sector Which is Focused on Injury and Recovery	Curative physical recovery trumps RTW	“When we provide acute care for someone, work is not our focus; it’s simply about restoring bodily functions.” (P19)
	Lack of integrated care	“If one thing isn’t going well, it’s the step after the rehabilitation center […] If I just look at how we’re [occupational physician] called in; we’re called in by HR, and if HR does not report it [RTW] then I don't know. And then it may well be that I see and speak to someone [patient] much later than desirable.” (P34)
		“It’s [occupational physician] not always seen as an equally attractive option by employees, so the added value that the occupational physician can offer is often… I think occupational physicians also really struggle with an image problem.” (P22)
	Provide timely and relevant RTW support to patient and work environment	“It’s a matter of translating what limitations someone has, what complaints someone has, what I recommend in terms of work. Sometimes I do this at the level of the disability, for example I advise not to bend or twist or climb, to name a few.” (P31)
		“Both in medical recovery and technically, you make the biggest steps in the first few months, including work reintegration. Anything that needs to happen after six months is just hard work for everyone. It becomes tough for the patient, the employee, but also for the manager.” (P18)
Motivation to RTW Depends on Patients’ Attitude Towards Abilities and Gains	Overestimate/underestimate own abilities	“I really have to take self-employed workers who do physically demanding jobs into account in my therapy. […] These are very challenging processes, so you have to sit down and explain the balance between load and load capacity, saying ‘This is where you want to go. I understand that you’re self-employed and want to improve, but look at what this is doing to your daily workload’.” (P29)
	Prolonged patient role hinders recovery and RTW	“In my experience, and what’s researched a lot, are the delaying effects of personal injury insurers on recovery. Patients unconsciously have an interest in maintaining their complaint patterns when there’s still a personal injury claim.”(P20) “I also see the other side: people who don’t place much importance on returning to work and instead want to take as much time as possible to recover thoroughly, focusing entirely on rehabilitation. For that latter group, we try to emphasize at a very early stage that regaining a societal role through work, whether paid or unpaid, strongly contributes to long-term well-being.” (P25)
	Patients’ motivation to return to meaningful work	“I see what it does for people when they get back into the swing of things, functioning in their preferred job, because they didn’t choose that job for nothing. They usually really enjoy it. The social contacts at work, contributing meaningfully to society, having your own daily structure. […] It has such a positive impact on people’s recovery.” (P18)
Importance of Receiving or Lacking Support From Employers and Colleagues		“It also strikes me that people who are in rehabilitation and experience a lot of support from colleagues are much more inclined to think about returning to work.” (P25)

#### Theme 1: Extent to Which the Injury Affects Work, Self-Confidence, and Finances

Depending on the type of injury, the execution of core tasks may be affected differently, if affected at all. According to HCPs, the injury was perceived to have a long-term impact on reintegration when patients sustained an injury that (temporarily) hindered execution of core tasks. Conversely, all HCPs perceived that the impact of the injury was minimized when patients had a job that was not greatly affected by the injury they sustained. For example, having a job that requires little physical activity and having a broken leg.

"Someone who has no physical job with a lower leg trauma may return to work more quickly.” (P36)

Also, HCPs mentioned that many self-employed patients were struggling with financial difficulties during recovery when facing long-term inability to work due to the injury. In general, HCPs perceived that without insurance, self-employed patients not only lost (part of) their income but also did not get treatment to avoid the high costs, and therefore had a slower recovery and RTW process. Furthermore, many HCPs mentioned that patients who had been unable to work for a while could feel insecure about their work abilities, and had worries whether they can perform on the same level as before their injury.

#### Theme 2: The (Lack of) Attention on Work in a Clinical Sector Which is Focused on Injury and Recovery

The perception of the involvement of HCPs and the degree to which they provide follow-up care in RTW after injury seems to influence the perception of their role in the RTW process.

*Curative Physical Recovery Trumps RTW* Professionals, particularly HCPs in the hospital, mainly focused on physical recovery and rehabilitation. Even though deemed important, RTW as a topic is deprioritized, especially immediately after sustaining the injury. In general, it was perceived that lack of time during consultations made all HCPs in the hospital prioritize what needs to be discussed, and as physical recovery is their core task, there is no time left for topics such as RTW.

“At first, if it’s a serious injury, you’re concerned with the recovery of that injury. Work and returning to society comes second. I often think this is something that could be discussed earlier in the guidance and treatment, and that’s a pity.” (P24)

Besides, HCPs working in hospitals tend to see RTW as the responsibility of professionals such as occupational physicians. These professionals are believed to have the necessary expertise to provide patients with appropriate guidance on RTW. Furthermore, HCPs mentioned that patients have the misconception that their physical complaints need to subside before they RTW, meaning patients postpone RTW discussions.

*Lack of Integrated Care* HCPs felt that the compartmentalization of the health care system and poor transferal between professionals prevented them from executing their work and to aid RTW. Occupational physicians for instance mentioned that rehabilitation centers oftentimes denied them access to patients, complicating timely discussions of RTW options.

“In an ideal world, you would know earlier what someone needs in the guidance and have more collaboration around a patient. Because it’s all about personalized care, everyone is different. […] Our system is not sufficient for that; it’s simply too bureaucratic and too compartmentalized.” (P24)

Furthermore, occupational physicians argued that patients perceive them to be biased in favor of the employer when it comes to RTW. For example, they felt patients perceived them to insist on working even when that would not be possible yet. Occupational physicians expressed they try to reassure patients by taking away any preconceptions during the first consultation, making clear that they are there for the patient.

"People often think that an occupational physician is a kind of referee, kicking people back to work, but that’s really not true. So I always try to take the elephant out of the room by explaining that first of all, I’m a doctor, which means that everything you say here falls under professional confidentiality […] And then the first relief has entered that room. Then you can do your job properly.” (P23)

*Provide Timely and Relevant RTW Support to Patient and Work Environment* HCPs expressed that they help patients’ RTW in various ways. For instance, by inventorying patients’ capacities and limitations in their job and workplace visitations by occupational therapists and physicians. Furthermore, psychological and emotional support is frequently offered not only by psychologists, but also by some physical therapists, particularly in preventing patients to RTW too fast and to not overstep physical boundaries. While most HCPs claimed they generally did not ask the patient about work, several HCPs explicitly mentioned they did do this, which helped them to provide support in RTW. Those HCPs also supported patients by referring to occupational physicians, physical therapists, and general practitioners.

“Are they self-employed or not? Do they have an office job or a physical job? So, I actually want to know fairly quickly: what is your relationship with your boss like? […] What does your work involve? […] to assess whether this might cause problems during the rehabilitation process.” (P29)

According to HCPs, the biggest progress in recovery is achieved in the first six months after sustaining the injury. The majority said that talking about RTW in an early stage after sustaining the injury benefits RTW. Depending on the severity of the injury, HCPs preferred to be involved early on in the patients’ recovery process to provide guidance in RTW. HCPs also expressed that receiving effective follow-up care right from the beginning can advance recovery, and therefore contribute to an earlier start of RTW.

“Well, what I try to do myself is very early refer to the occupational physician, because I think that he or she must have contact very early on. So that, even if [the patient] can’t do anything yet, at least there should be contact [and discuss], when can we pick that [work] up again and what alternative things can we do.” (P27)

#### Theme 3: Motivation to RTW Depends on Patients’ Attitude Towards Abilities and Gains

According to HCPs, the RTW process seemed to be influenced by how motivated patients are to RTW. That is, patients not only need a desire to RTW but also need to RTW at a pace that is appropriate for their circumstances.

*Overestimate/Underestimate Own Abilities* Many HCPs mentioned that patients risk obstructing recovery and RTW when they overestimate or underestimate their abilities after sustaining an injury. According to HCPs, oftentimes this overestimation can be found in self-employed patients. In contrast, other patients can be anxious to “overdo it” and therefore delay their (full) RTW. In such situations, HCPs needed to encourage patients to take one step at a time, so that they gradually gain confidence in their own abilities.

“I try to help people build up best as possible within their limits, because as a psychologist you often see that people sometimes want too much or have deprived themselves of recovery due to overload for a long time. Sometimes we have to tone down a bit and say just take it step by step. Sometimes, in contrast, we have to break through fear.” (P21).

*Prolonged Patient Role Hinders Recovery and RTW* Some HCPs perceived that patients lacked commitment and a sense of responsibility for their recovery process, and therefore in RTW. These HCPs explained they wanted to support patients in their recovery when patients are committed to treatment and adhered to prescribed exercises for instance. They felt that some patients became dependent on the support system around them, expecting that professionals would make recovery happen without putting in much effort themselves. Also, according to HCPs, when patients have pending personal liability cases, they sometimes believe they will get financial compensation for their injuries and potential loss of income for instance. To get compensated, patients sometimes over-emphasize their symptoms, and subsequently unnecessarily delay RTW.

“Some people clearly have a hidden agenda, like ‘you don’t really want to recover. You think it’s in your best interest to stay sick for as long as possible because you’re hoping to get a large compensation’ or something like that.” (P23)

*Patients’ Motivation to Return to Meaningful Work* Most HCPs believe that patients’ motivation is essential in RTW. They experience that some patients want to RTW because work provides meaning, and they want to feel part of society again. Additionally, motivation to RTW is perceived to be influenced by how much someone likes their job, and how supported someone feels by colleagues. According to HCPs, those patients actively look for information and guidance in RTW, and are more likely to return sooner.

“We actually encounter clients who simply want to return to society and return to a sense of meaning: I matter.” (P20)

#### Theme 4: Importance of Receiving or Lacking Support From Employers and Colleagues

The RTW process seemed to be influenced by whether colleagues and employers are understanding and supportive during reintegration or not.

Receiving no support from work relations was thought to negatively impact patients’ RTW intentions. Besides lack of contact during rehabilitation, HCPs mentioned that perceived work expectations from colleagues could be hindering for patients. For instance, colleagues were perceived to lack sympathy and understanding when they were not informed about the reintegration plan, or when patients seemed fine but still worked less because of latent symptoms. This seemed to be feared by TBI patients specifically. HCPs felt that TBI patients are insecure about communicating such latent symptoms and prognoses to their work relations. Otherwise, HCPs expressed that colleagues have the tendency to overly protect patients by encouraging them to take it easy, which could be perceived as supportive but in fact can impede reintegration and therefore does not support RTW.

“Colleagues either say [to the patient] ‘calm down, go home, you don't have to come, we will solve it’. They actually hinder reintegration. Or they say ‘yeah, then Í have to work harder’ and then the overasking starts.” (P34)

In contrast, HCPs expressed that receiving support from colleagues or employers helps patients in RTW. Support could entail small-talk with colleagues during rehabilitation, or concrete support like arranging transportation to get to work. Such supportive actions made the patient feel welcome and wanted in the workplace.

## Discussion

This qualitative study aimed to identify and gain deeper insights into the barriers and facilitators in RTW after physical injury by combining the perspectives of a heterogeneous group of trauma patients and HCPs from diverse fields. Overall, work seemed to be an important topic to patients and HCPs. Most importantly, the extent to which core work tasks were or were not affected by the sustained injury seemed to hinder or facilitate RTW across injuries. That is, if the type of injury has direct impact on the ability to execute tasks that are at the core of one’s job, this would hinder the ability to RTW. In addition, receiving timely and personalized RTW support and follow-up care by HCPs was perceived as facilitating successful RTW.

Our results further show that patients and HCPs share similar ideas on the barriers and facilitators in RTW after injury. Findings resonate with previous research, which found that RTW could be impacted by (a lack of) work adjustments [16, 17, 35–37], (a lack of) support in the workplace [13, 16, 17, 26, 36–38], and being motivated to RTW [17, 37, 38]. Commonalities relating to perceived barriers for sustainable RTW were found in (1) limited consultation time to discuss RTW with HCPs in hospital, (2) lack of focus on RTW follow-up care during the rehabilitation process, and (3) patients receiving limited support or sympathy from the work environment. Regarding facilitating factors in RTW, similarities were related to (1) core work tasks not being affected by the injury, (2) the importance of patients receiving well-timed, relevant support from HCPs, and (3) patients receiving support from their work environment.

Despite the similarities in perceptions of barriers and facilitators in RTW, some differences in perceptions between patients and HCPs can be observed. While there seems to be mutual agreement that support in RTW from HCPs is necessary, the perspectives of patients and HCPs primarily diverged on the barriers related to follow-up care by HCPs in hospital specifically. HCPs in hospital recognize that work is important to patients. However, they feel that with the limited consultation time they have, which is a frequently mentioned barrier in trauma care and other medical contexts [39–41], their main focus should be on functional (physical) recovery. They expect other parties to take over after hospital discharge on aspects such as RTW. In addition, HCPs other than occupational physicians and other reintegration professionals may have the feeling they lack necessary skills to discuss RTW. Conversely, similar to Roodbeen et al. [13], patients indicated the desire to use (part of the) hospital consultations to discuss issues outside of physical recovery, such as RTW. They experience the focus of HCPs on physical well-being to overshadow life after injury, and therefore perceive follow-up care by HCPs in hospital on RTW to be insufficient.

For practice, overarching factors across injuries are of added value as interventions could be developed that are applicable to a larger trauma population instead of specific populations only. For instance, it would be important to manage patients’ expectations regarding follow-up care in RTW provided by HCPs in hospital. They could refer patients to beneficial resources for information on RTW after injury, for instance to HCPs who focus on this aspect (e.g., an occupational physician or occupational therapist), but also to online resources provided by the hospital offering specific RTW information. Implementation of a readily available (digital) tool, like a digital health application, offering this information may be beneficial. These applications have the potential to convey timely, personalized, and reliable health information which is always accessible [42] and have been found to be useful in providing rehabilitation support to TBI patients [43]. It is therefore not unthinkable that such applications are beneficial to other trauma patients as well. Additionally, applications allow for information to be personalized, meaning that information can be provided that is applicable to an individual patient [44]. Future research could explore what patients need in follow-up care provision to successfully RTW after injury, and what HCPs need to provide adequate care to those patients. Furthermore, research should focus on how these needs can be translated into a digital health application. And in a broader light, in agreement with Cancelliere et al. [27], it could be explored whether these *transfactors* in RTW apply to patients with other illnesses.

Strengths of this study lie in the inclusion of multiple stakeholders and the focus on the overarching factors in RTW after trauma across a variety of injuries. Our study underscores the importance of including various perspectives and injuries, as some viewpoints differed between patients and HCP and barriers went beyond functional impairments, as the nature of the work and personalized support in RTW seemed to be a more fundamental factor in RTW than the injury itself. A few limitations of this study do need to be acknowledged. First, most patients sustained their injury during or a few months prior to the COVID pandemic. During the pandemic, HCPs had to prioritize or alter their care provision, meaning that non-life threatening cases were temporarily put on hold and care was provided at a distance if possible. This may have negatively influenced patients’ perceptions of the care provided by HCPs. Second, selection bias could have taken place, as the recruited patients who were dissatisfied with the follow-up care provision may have been more motivated to participate in the study. Lastly, health care professions were not equally represented, possibly giving more weight to information coming from professions that were overrepresented, and less weight to underrepresented professions. For instance, only one general practitioner was included, opposed to four occupational physicians. This imbalance, however, does not diminish the importance and value of the information that came from these health care professions.

## Conclusion

This study explored what trauma patients and HCPs perceive to be barriers and facilitators to RTW after physical injury across injuries. RTW seems to be hindered or facilitated by the extent to which core work tasks were or were not affected by the sustained injury. Additionally, while views of patients and HCPs mostly aligned, their perspectives differed on the follow-up care provision by HCPs in hospital. It is advised to ask patients about their work to provide adequate follow-up care focused on RTW. To manage patients’ expectations, more focus on personalized information about RTW provided by the hospital could be useful. Future studies could explore what the information needs are and how these can be implemented in a digital health application.

## Data Availability

No datasets were generated or analyzed during the current study.

## Appendix 1: Topic Guide Patients

*Moderators welcome everyone and introduce themselves*

**Background**

Research shows that a large portion of people who sustain physical injuries experience difficulties in their daily lives and are unable to work for an extended period, with some not returning to work at all even after 2 years. However, it remains unclear what exact challenges individuals face when attempting to return to work and what support and guidance they need in this process. Additionally, work is perceived as an important part of recovery by many patients.

**Objective**

The goal of these group discussions/this interview is to gain insight into the experiences of patients who have suffered injuries regarding returning to work, as well as to understand their needs for support and guidance in this regard. We appreciate your willingness to participate in this. We have invited individuals who have sustained physical injuries up to 5 years ago. You are gathered here today as a group/You are interviewed today to discuss your experiences, needs, and opinions regarding returning to work and the follow-up care and support involved.

**What does the meeting look like?**

The meeting will last approximately 1.5 h/1 h. We will ask you various questions and would like to hear your answers, allowing you to also respond to each other. It is important to know that you are free to share whatever you want, and there are no right or wrong answers. It is about what you think and why you think it. It is not necessary for you to agree with each other. It is crucial that all different opinions, if any, are expressed. Everyone will have the opportunity to share their opinion. Please try not to talk over each other.

We may interrupt you. This does not mean we are not interested in what you have to say. However, we want to ensure that everyone has a chance to speak.

In case of technical issues, you can use the chat or call the researcher [phone number].

**Anonymity and independence**

The group discussion will be recorded in Microsoft Teams, as indicated in the information letter, and will be transcribed verbatim. We do this only to process the data accurately. The conversation transcript will not contain your name, and the data will not be traceable back to you as an individual.

If you wish to speak, you can take the floor or raise your virtual hand, see the symbol at the bottom of the screen. Clicking on the hand icon will raise your virtual hand. Clicking on it again will lower your hand. Please turn on your camera and mute your microphone when you are not speaking.

**Questions?**

Do you have any questions before we begin? We will now start the Teams recording.

*Introduction*

Before we officially begin, we will start with introductions. Could you briefly state who you are, where you work, and what your role is? Additionally, we would like to know why you are participating in this focus group.

*Topic 1: Experiences with maintaining, losing, and/or returning to work*

1. *What impact did the injury have on your work immediately after the injury?*
  - (Temporarily) stopped working/continued doing the same job/switched to another job
  - Voluntarily stopped/switched to another job or forced to do so due to injury
  - Job arrangement: hours/tasks/functioning/responsibilities
  - Job satisfaction
  - Work ethic/change in the value of work?
  - Relationships with others
  - Expectations from surroundings
  - Expectations from yourself
2. *What impact did the injury have on your work in the long term?*
3. *What impact does the injury still have on your work now?*
  - Job arrangement: hours/tasks/functioning/responsibilities
  - Job satisfaction
  - Work ethic/change in the value of work?
  - Relationships with others
  - Expectations from surroundings
  - Expectations from yourself
4. *How did you deal with the changes in the work domain?*
5. *To what extent are you currently able to achieve what is important to you in your work?*
  - What does work mean to you?
6. *What concerns and doubts do you have for the future regarding work?*
7. *What hopes and dreams do you have for the future regarding work?*
8. *What do you need to (further) improve your functioning at work?*
9. *To what extent did the work environment help or hinder you in returning to work?*
  - How was the contact with the supervisor? Were you supported? Were your needs considered?
  - How was the contact with colleagues?
  - How was the collaboration with colleagues? And with HR?
  - Were work adjustments/task content adjustments necessary?

*Topic 2: Experiences with and needs for follow-up care and guidance in* *(return to) work*

10. *What* *follow-up care* *and guidance did you receive in the field of work?*
  - Within ETZ: from the doctor, nurse, physiotherapist, psychologist
  - Outside ETZ: from the occupational physician, general practitioner, (occupational or primary care) psychologist, social work, primary care physiotherapist, etc.
  - From the work environment
11. *What did you perceive as positive or useful in the* *follow-up care* *when returning to work? Why was this useful? What did it yield?*
12. *What did you miss in* *follow-up care* *or guidance when returning to work?*
13. *What would optimal* *follow-up care* *and guidance look like according to you?*
  - What should this focus on?
  - Does it help if the doctor asks about your work? In what way could this be a topic of conversation?
  - When should this be provided? (immediately after the injury/later)
  - By whom should this be provided/offered?
  - In what form could this be provided/offered?

*Ending*

This was the last question of the focus group/interview, we are concluding now. Thank you very much for your participation! A lot of relevant information has been brought forward!

Is there anything else you would like to share? A final message? If anything else comes to mind later, feel free to email us.

We have several focus groups and interviews. Once these are completed, we will analyze the conversations. We will keep you informed about the results of this research. Once again, thank you for your participation, the token of appreciation will be sent to you via email.

## Appendix 2: Topic Guide Health Care Professionals

*Moderators welcome everyone and introduce themselves*

**Background**

Injuries resulting from accidents have a significant impact on the lives of patients and their loved ones. Recent research has shown that regardless of the type or severity of an accident, injuries affect various aspects of the lives of trauma patients. Patients experience difficulties in participating in daily activities that are important for feeling satisfied with their lives. Additionally, work is seen as an important part of recovery by many patients.

**Objective**

The aim of these group discussions is to gain insight into the experiences and roles of various health care professionals in the care of trauma patients and the support and guidance in returning to work. We greatly appreciate your participation in this. You have come together as a group to discuss your experiences in guiding trauma patients in returning to work and what your role in this is or could be.

**What does the meeting look like?**

The meeting will last approximately 1.5 h. We will ask you various questions and would like to hear your answers, allowing you to also respond to each other. Everyone is free to say what he/she wants, and there are no right or wrong answers. It is not necessary for you to agree with each other. It is important that all different opinions, if there are any, are expressed. Everyone will have the opportunity to share their opinion. Please try not to talk over each other.

We may interrupt someone. This does not mean we are not interested in what you have to say. However, we want to ensure that everyone has a chance to speak.

In case of technical issues, you can use the chat or call the researcher [phone number].

**Anonymity and independence**

The group discussion will be recorded in Microsoft Teams, as indicated in the information letter, and will be transcribed verbatim. We do this only to process the data accurately. The conversation transcript will not contain your name, and the data will not be traceable back to you as an individual.

If you wish to speak, you can take the floor or raise your virtual hand. Clicking on the hand icon will raise your virtual hand. Clicking on it again will lower your hand. Please turn on your camera and mute your microphone when you are not speaking.

**Questions?**

Do you have any questions before we begin? We will now start the Teams recording.

*Introduction*

Before we officially start, let us begin with introductions. May I ask you to briefly state who you are, where you work, and what your role is? We'd also like to hear why you are participating in this focus group.

*Topic 1: Care, follow-up care, or guidance of trauma patients*

1. *What is your role in the care, follow-up care, or guidance of trauma patients?*
2. *How do you support patients in the process of returning to work?*
  - What does this guidance entail?
  - When does this guidance take place? (immediately/some time after injury/long time after injury?)
  - Can you provide examples of assistance requests regarding Return to Work (RTW) from patients?
  - Do you have enough time and resources to guide patients with these assistance requests?
3. *How do you support patients who have already returned to work in the workplace?*
  - What does this guidance entail?
  - When does this guidance take place? (immediately after injury/some time after injury/long time after injury?)
  - Can you provide examples of work-related assistance requests from patients?
  - Do you have enough time and resources to guide patients with these work-related assistance requests?
4. *What do you observe from your own practice that works well in guiding patients in returning to work?*
5. *What do you observe from your own practice that could be improved in guiding patients in returning to work?*
  - Why are these improvements not currently implemented in practice?
6. *What would optimal follow-up care and guidance in returning to work for trauma patients look like according to you?*
  - When should the guidance start? (immediately/during treatment/afterwards)
  - By whom should this be provided/offered?
  - In what form should this be provided/offered?
7. *What do you need to be able to provide this follow-up care or guidance?*

*Topic 2: Experiences of trauma patients in returning to work according to health care providers*

8. *How does the return to work process typically unfold for most of your patients?*
  - What are the reasons behind this?
  - What is positive or going well? (specific examples)
  - What could be improved? (specific examples)
  - Role of the work environment (supervisor, colleagues, workplace itself)
  - What are reasons why patients successfully return to work?
  - What are reasons (besides physical inability to work) why patients cannot successfully return to work?
9. *What challenges do patients encounter when returning to work?*
  - Job arrangement: hours/tasks/functioning/responsibilities
  - Job satisfaction
  - Work ethic/change in the value of work?
  - Relationships with others
  - Expectations from surroundings
  - Expectations from oneself
  - Role of the work environment
10. *What do patients need according to you in order to return to work?*

*Ending*

This was the last question of the focus group, we are concluding now. Thank you very much for your participation! A lot of relevant information has been brought forward!

If anything else comes to mind later, feel free to email us. We have several focus groups. Once these are completed, we will analyze the conversations. We will keep you informed about the results of this research. Once again, thank you for your participation, and for those who have indicated they would like to receive a token of appreciation: it will be sent out during the week!
